# Short‐term effects of fresh mother's own milk in very preterm infants

**DOI:** 10.1111/mcn.13430

**Published:** 2022-09-13

**Authors:** Jing Huang, Zhi Zheng, Xiao‐yan Zhao, Li‐han Huang, Lian Wang, Xiao‐lan Zhang, Xin‐zhu Lin

**Affiliations:** ^1^ Department of Neonatology, Xiamen Maternal and Child Care Hospital, Women and Children's Hospital, School of Medicine Xiamen University Xiamen China; ^2^ Department of Neonatology Xiamen Humanity Hospital Xiamen China

**Keywords:** breastfeeding, infant, premature, prognosis, very preterm infant

## Abstract

Fresh mother's own milk (MOM) can protect preterm infants from many complications. Often MOM is pasteurized for safety, which can deactivate cellular and bioactive components with protective benefits. Questions remain regarding whether pasteurized MOM provides the same benefits as fresh MOM. The aim of this study was to evaluate the association and feasibility of feeding very preterm infants with fresh MOM. This prospective cohort study included 157 very preterm infants born before 32 weeks' gestational age and with a birthweight below 1500 g. Of these, 82 infants were included in the fresh MOM without any processing group and 75 infants were included in the pasteurized never‐frozen MOM (PNFMOM) group. The mortality rate, survival rate without severe complication, incidence of complications, feeding indexes and growth velocities were compared to assess the association and feasibility of feeding fresh MOM. Compared with the PNFMOM group, the fresh MOM group had a higher survival rate without severe complications (*p* = 0.014) and a lower incidence of bronchopulmonary dysplasia (*p* = 0.010) after adjustment for confounders. The fresh MOM group regained birthweight earlier (*p* = 0.021), reached total enteral feeding earlier (*p* = 0.024), and received total parenteral nutrition for less time (*p* = 0.045). No adverse events associated with fresh MOM feeding were recorded. Feeding fresh MOM may reduce the incidence of complications in very premature infants. Fresh MOM was shown to be a feasible feeding strategy to improve preterm infants' outcomes.

## Key messages

- Very few studies have directly compared morbidity and mortality between preterm infants fed fresh mother's own milk and pasteurized mother's own milk.
- An increased survival rate without severe complications and decreased risks for bronchopulmonary dysplasia were found in premature infants fed with fresh mother's own milk without any processing compared with pasteurized never‐frozen mother's own milk. This may be taken into consideration when debating whether or not to pasteurize mother's own milk to inactivate cytomegalovirus.
- Feeding fresh mother's own milk was found to be a feasible feeding strategy to improve preterm infants' outcomes.

## Graphical abstract

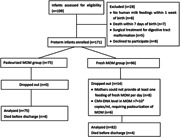

## 1 BACKGROUND

Breast milk is recognized as the ideal feeding type for all infants due to its known nutritional components (e.g., proteins, carbohydrates, vitamins and minerals) and bioactive components (e.g., live immune cells, lactoferrin, secretory immunoglobulin A, cytokines, hormones and growth factors) that possess immune and antimicrobial activities (Ballard & Morrow, 2013; Mosca & Giannì, 2017). Multiple studies have suggested that breastfeeding in the neonatal intensive care unit (NICU) can improve infants' feeding tolerance (Assad et al., 2016); decrease the incidence of late‐onset sepsis (LOS) (Cortez et al., 2018), necrotizing enterocolitis (NEC) (Maffei & Schanler, 2017; Miller et al., 2018) and retinopathy of prematurity (ROP) (J. Zhou et al., 2015); and improve neurodevelopmental outcomes (Belfort et al., 2016). However, the current breast milk feeding protocols for hospitalized premature infants in China are far from satisfactory. A multicenter investigation conducted in 2012 found that only 2 of 15 (13%) tertiary neonatal care centres in China use breast milk as the source of enteral feeding for preterm infants with birthweight <1500 g, and the overall proportion of breast milk fed to these infants was less than 10% in these two hospitals (The National Cooperation Group on Nutrition, The Chinese Neonatologist Association, 2015). A study conducted in 29 hospitals found that the proportion of mother's own milk (MOM) fed to preterm infants was only 37.2%, and donated breast milk or formula was given when the amount of available MOM was insufficient (Jiangsu Multicenter study Coordination Group for breast milk Feedings in Neonatal Intensive Care, 2018). Thus, collection of MOM, with appropriate screening, and pasteurized donor human milk (DHM) are the next best choices, with the last option being preterm formula (PF) when MOM and DHM are not available. Currently, the lack of uniform standards in the management of preterm infant breastfeeding, the limited space in the NICU and insufficient staffing and inadequate knowledge and technical capabilities of physicians hinder the implementation of breastfeeding for preterm infants in China. Common storage methods for breast milk include refrigeration at a temperature of 4°C or freezing at a temperature of −18°C or lower, and in some NICUs, breast milk is pasteurized to prevent cytomegalovirus (CMV) infection in very preterm infants (Yang & Lu, 2020). Numerous studies have revealed that pasteurization and frozen storage decrease the macronutrient and immunoglobulin compositions of breast milk to varying degrees, with reductions in protein, fat, carbohydrates, energy content, secretory immunoglobulin A, lactoferrin and antioxidants (Adhisivam et al., 2019; Chang et al., 2013; Orbach et al., 2019). In addition, defrosted human milk does not contain any live stem cells, because these cells have a half‐life of approximately 4 h and die when the human milk is frozen and/or heated (Hassiotou et al., 2013).

Studies with different designs have compared the effects of MOM, donor milk, and PF in preterm infants and reported highly variable outcomes. Very few studies have directly compared neonatal morbidity and mortality between infants fed fresh human milk and pasteurized MOM, and thus, we performed a single‐centre prospective cohort study to evaluate the association and feasibility of feeding very preterm infants (gestational age <32 weeks and birth weight <1500 g) fresh MOM within 3 h of expression.

## 2 METHODS

### 2.1 Participants

Infants born before 32 weeks of gestational age and with a birth weight below 1500 g between 1 June 2018 and 31 May 2020 who were admitted to the NICU of Xiamen Maternal and Child Care Hospital within 12 h of birth were eligible for this study if the parents gave written informed consent. Because the study started shortly after birth, informed consent was obtained before delivery, if possible. The exclusion criteria included major congenital anomalies or birth defects, death within 1 week of birth, or any maternal or infant condition that prevented the administration of MOM within 1 week after birth, such as contraindications of breast milk feeding. The duration of the study was from enrolment to 36 weeks of gestational age or to discharge from the NICU, whichever occurred first. Infants who were discharged before 36 weeks' corrected gestational age were followed up until they reach 36 weeks' corrected gestational age. The ethics committee of Xiamen Maternal and Child Care Hospital for human research approved this study (approval no. KY‐2016‐020), and the parents of all study participants provided written informed consent. All procedures involving human participants were performed in accordance with the ethical standards of the institutional and national research committee and with the 1964 Declaration of Helsinki and its later amendments or comparable ethical standards.

### 2.2 Feeding protocol

At the time of recruitment, the infants were assigned to two groups according to whether the mothers agreed to provide fresh MOM. The infants whose mothers committed to providing fresh MOM within 3 h of expression without cooling, freezing, pasteurizing or heating were included in the fresh MOM group. The infants whose mothers did not agree to provide fresh MOM and did agree for their infants to receive pasteurized never‐frozen MOM (PNFMOM) were included in the PNFMOM group. Further, we also stratified the infants in the fresh MOM group according to the proportion of fresh MOM given in the enteral feedings. If maternal milk was insufficiently available, PF was used as a supplement. No donor milk was used.

During the 2 years of the study, we followed the same feeding protocol. Following standard procedures, all infants without contraindications for enteral feeding were started on enteral feeding as early as possible through a nasogastric tube and were provided total parenteral nutrition (TPN) within 24 h after birth. Feedings were initiated at 10 ml/kg/day and then advanced daily by 15–20 ml/kg as tolerated after 3 days. Powdered cow's milk‐based human milk fortifiers were started when oral feeding reached 100 ml/kg/day. Vitamin D 800 IU/day was added as soon as enteral nutrition was well tolerated. Parenteral support was stopped when oral feeding reached 130 ml/kg/day.

### 2.3 MOM collection

The fresh MOM was stored in a sterile plastic container and used for feeding within 3 h of expression at the beginning. In the later stage, mothers were encouraged to present in the NICU to pump fresh human milk every day, 7 days a week. For the PNFMOM group, MOM was transferred to a sterile plastic container and stored in the refrigerator (4°C). Within 12 h, the milk was pasteurized at 62.5°C for 30 min (Adhisivam et al., 2019). The PNFMOM was then stored in the refrigerator and rewarmed before feeding. The same standard processing procedures of pasteurization were applied for any milk expressed by the mother that could not be used within 3 h after expression.

All containers were labelled with the infant's bar code and given to a research nurse who input the details of the milk and feeds into a database and prepared the milk and delivered the milk to the nurse caring for the baby. Infants were fed by the infants' nurses. The study was double‐blinded and the clinicians and nurses caring for the infants were blinded to the group allocation.

### 2.4 Safety

In accordance with the Data Safety and Monitoring Committee, (1) all premature infants were routinely screened for urine CMV DNA within 2 weeks after birth and before discharge (Luck et al., 2017). If a positive result was obtained, then a serological examination was conducted. Human milk was monitored for CMV‐DNA at weeks 2, 4 and 6 after expression. The risk of CMV infection significantly increased if the CMV‐DNA level in human milk was greater than 7 × 10^3^ copies/ml (van der Strate et al., 2001). Therefore, if the CMV‐DNA level was less than 7 × 10^3^ copies/ml, we continued to feed the infant fresh MOM. However, if the CMV‐DNA level was above this level, the human MOM was pasteurized and the infant was dropped from the fresh MOM group. (2) The feeding protocol was stopped if NEC occurred. For infants in both groups, once intestinal function recovered, we restarted the feeding protocol. (3) Lessons on hand hygiene along with the expression, collection, storage, transport and administration of MOM were provided to medical staff and parents to ensure that the MOM was not contaminated, and critical incidence reports such as missed feedings, infants receiving the wrong milk and any other incident related to feeding or treatment were monitored.

### 2.5 Measurement

Maternal and infant demographic and clinical data were collected prospectively. Small for gestational age (SGA) (<10th percentile) (Villar et al., 2014), intraventricular haemorrhage (IVH) ≥ Grade 2, and periventricular leukomalacia were defined as described by Flores et al. (2020) and Hinojosa‐Rodríguez et al. (2017).

The primary outcomes were the incidence of mortality and survival without severe complications. Severe complications included NEC of at least Stage 2, LOS, moderate‐to‐severe bronchopulmonary dysplasia (BPD) and ROP requiring treatment. The secondary outcomes were the incidence of NEC, LOS, BPD and ROP; feeding indexes and growth velocities; and the feasibility of fresh MOM. NEC was defined as NEC greater than Stage 2 according to Bell's Staging Criteria (Bell et al., 1978). LOS was defined by a positive blood culture collected 72 h after birth. BPD was defined as the need for >21% supplemental oxygen for >28 days, and moderate‐to‐severe BPD was defined as oxygen treatment for at least 28 days along with the need for supplemental oxygen or positive pressure ventilation at 36 weeks' postmenstrual age or discharge for home, whichever came first (Abman et al., 2017). ROP was defined according to the international classification of ROP (International Classification of Retinopathy of Prematurity, 2005). Intervention for ROP was defined as surgery (laser or cryocoagulation) or the application of bevacizumab. Feeding indexes included time to regain birth weight, time to achieve total enteral feeding (150 ml/kg/day) (Dutta et al., 2015), duration of TPN (TPN is ordinarily discontinued when enteral feeding reaches 130ml/kg/day), and parenteral nutrition‐associated cholestasis (PNAC), (Wang et al., 2020) and feeding intolerance (Evidence‐Based Medicine Group, Neonatologist Society, Chinese Medical Association, 2020), which was assessed by multiple factors: gastric residual volume of more than 50% of the previous feeding volume; emesis or abdominal distention or both; and a decrease, delay or discontinuation of enteral feedings. Growth velocities included weight growth rate [g/(kg·day)], head circumference (HC) growth rate (cm/week) and length growth rate (cm/week). The weight growth rate after the recovery of birth weight was calculated as [1000 × ln (discharge weight/birth weight)]/(discharge age − return to birth weight age) (Patel et al., 2009). The HC growth rate was calculated by subtracting the HC at birth from the HC at discharge and dividing it by the length of stay (weeks). The length growth rate was calculated by subtracting the length at birth from the length at discharge and dividing it by the length of stay (weeks).

### 2.6 Statistical analyses

For the feasibility evaluation, the rate of recruitment and rate of completing the study in the fresh MOM group were analysed. For the safety evaluation, the critical incidence reports of missed feeding, infection related to feeds and CMV infection were reviewed (Rawlinson et al., 2017).

SPSS Statistics for Windows, Version 25.0 (IBM Corp.) was used to perform all statistical analyses. The baseline demographics, primary outcomes and secondary outcomes were presented using descriptive methods. Qualitative variables were compared using the *χ* ^2^ test. Numerical variables were compared using an independent‐sample *t*‐test or a nonparametric test (Mann–Whitney *U*‐test). Univariate analyses and multivariable logistic regression analyses were performed to assess the association between the type of MOM and neonatal mortality and morbidity after identifying potential confounders. Differences were deemed statistically significant if *p* < 0.05.

## 3 RESULTS

### 3.1 Baseline characteristics

**Figure 1:**
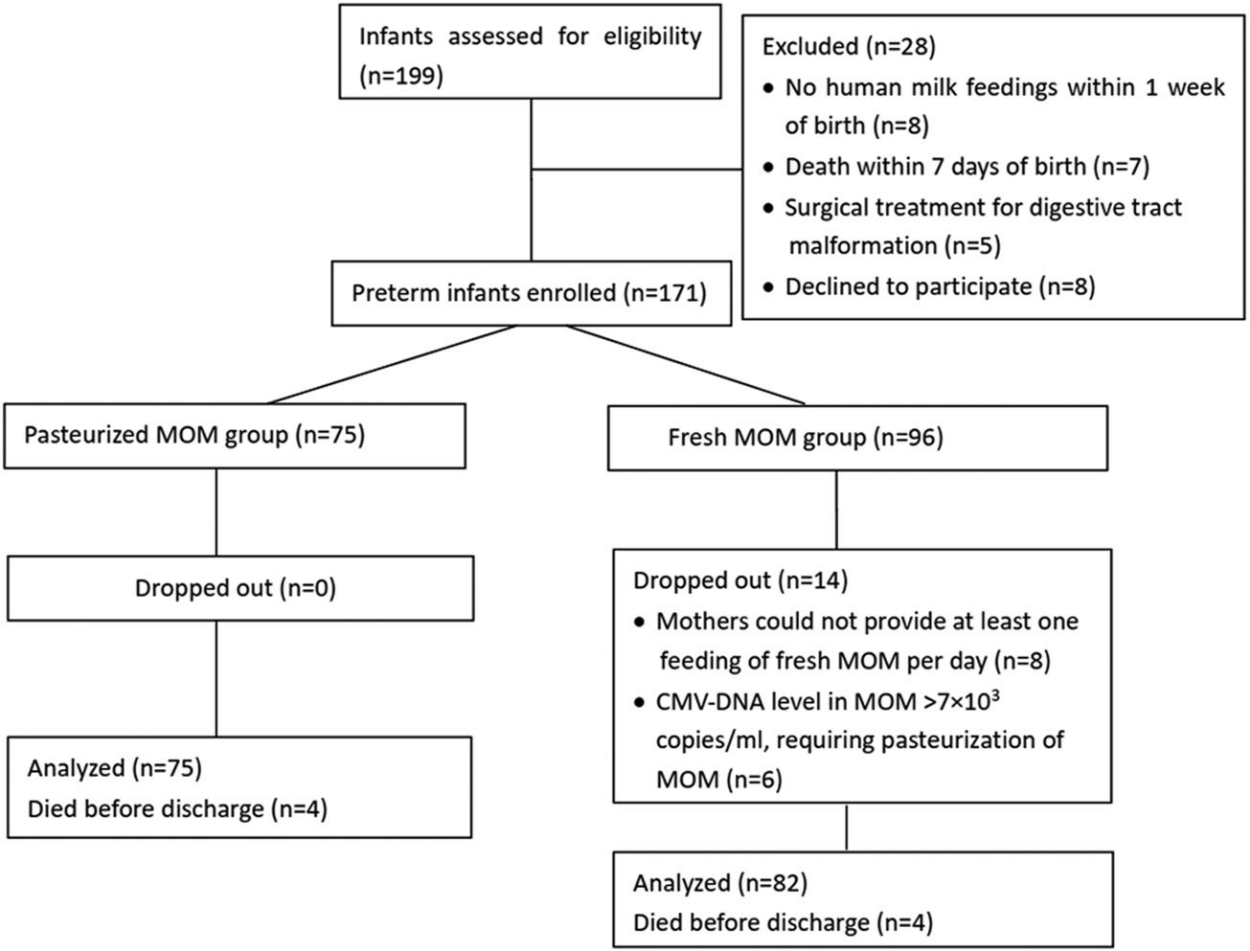
Flowchart of patient inclusion. CMV, cytomegalovirus; MOM, mother's own milk.

Between 1 June 1 2018 and 31 May 2020, 199 preterm infants born before 32 weeks' gestational age with a birth weight less than 1500 g were assessed for eligibility, of whom 157 infants (82 infants in the fresh MOM group and 75 infants in the PNFMOM group) were included in the final analysis (Figure 1). No differences in maternal and infant characteristics were detected between the fresh and PNFMOM groups (Table 1).

**Table 1:** Baseline characteristics of the study population

Variable	Fresh MOM group (*n* = 82)	PNFMOM group (*n* = 75)	*t*/*χ* ^*2*^/*Z* value	*p* Value
Maternal factors				
Caesarean delivery, *n* (%)	56 (68.3)	42 (56.0)	2.523	0.112
Hypertension, *n* (%)	14 (17.1)	8 (10.7)	1.334	0.248
Gestational diabetes, *n* (%)	29 (35.4)	32 (42.7)	0.879	0.349
ROM > 18 h, *n* (%)	19 (23.2)	19 (25.3)	0.100	0.752
Clinical chorioamnionitis, *n* (%)	4 (4.9)	2 (2.7)	0.093	0.760
Any antenatal corticosteroid, *n* (%)	74 (90.2)	69 (92.0)	0.149	0.700
Infant factors				
Birth weight (g), mean ± SD	1189 ± 195	1149 ± 207	1.248	0.214
Gestational age (weeks), mean ± SD	29.3 ± 1.4	29.1 ± 1.5	0.989	0.324
Male sex, *n* (%)	47 (57.3)	39 (52.0)	0.447	0.504
SGA (<10th percentile), *n* (%)	10 (12.2)	8 (10.7)	0.090	0.764
Apgar scores, M (IQR)				
1 min	8 (7, 9)	8 (7, 8)	1.361	0.173
5 min	9 (8, 9)	9 (8, 9)	0.793	0.428
10 min	9 (9, 9)	9 (9, 9)	0.189	0.850
Surfactant administration, *n* (%)	57 (69.5)	54 (72.0)	0.117	0.732
CPAP (days), mean ± SD	14.6 ± 6.3	16.6 ± 6.9	1.961	0.052
Mechanical ventilation (days), M (IQR)	0 (0, 2)	1 (1, 2)	0.518	0.605
Oxygen (days), M (IQR)	26 (23, 27)	26 (23, 33)	0.808	0.419
IVH ≥ Grade 2, *n* (%)	20 (24.4)	25 (33.3)	1.532	0.216
PVL, *n* (%)	5 (6.1)	7 (9.3)	0.581	0.446
Proportion of MOM intake (%), M (IQR)	96 (83, 100)	90 (80, 100)	1.220	0.223

Abbreviations: CPAP, continuous positive airway pressure; IVH, intraventricular haemorrhage; M (IQR), median (interquartile range); MOM, mother's own milk; NEC, necrotizing enterocolitis; PNFMOM, pasteurized never‐frozen MOM; PVL, periventricular leukomalacia; ROM, rupture of membranes; SD, standard deviation; SGA, small for gestational age.

**Figure 2:**
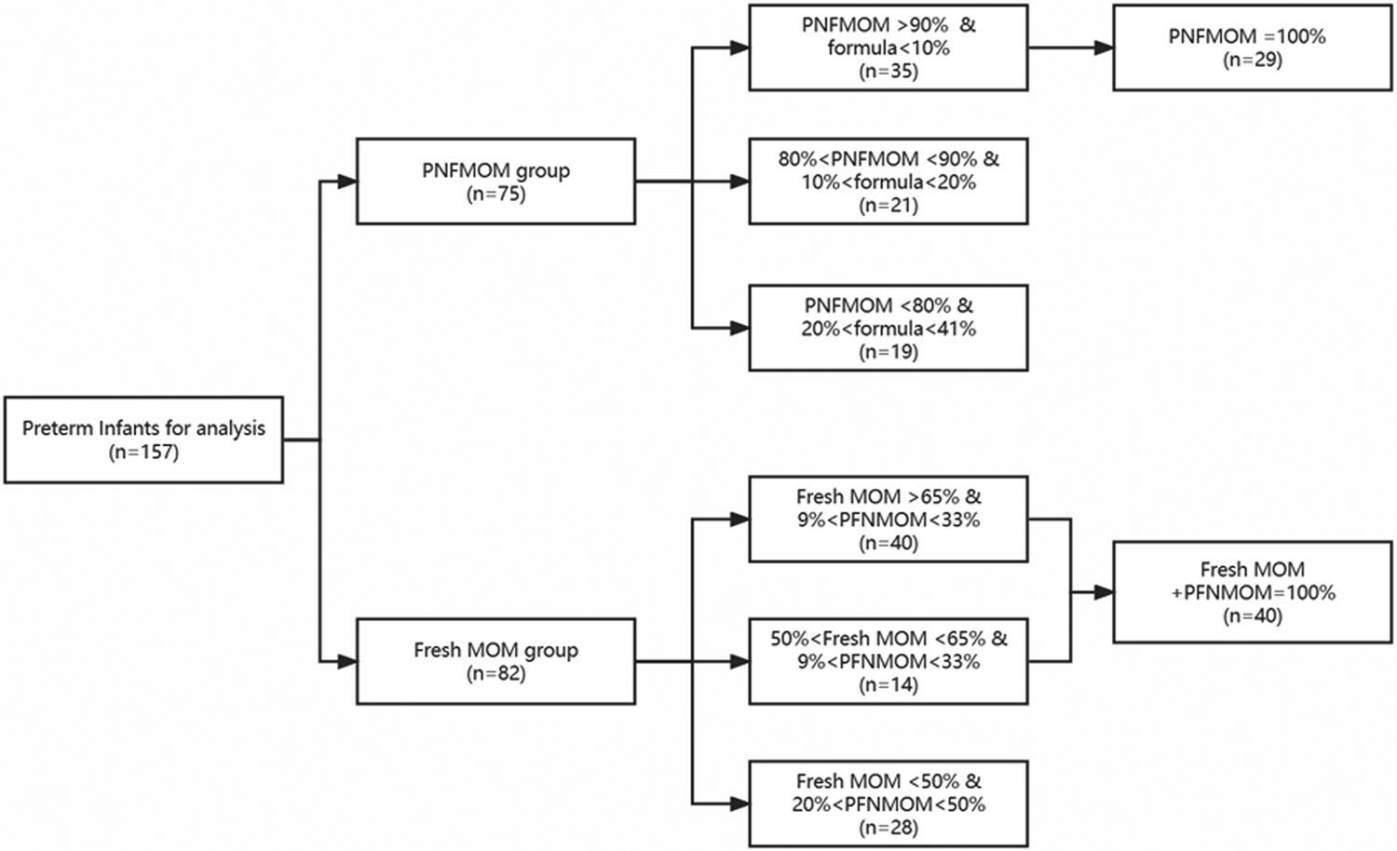
Infant number of different MOM in the fresh MOM and PNFMOM groups. MOM, mother's own milk; PNFMOM, pasteurized never‐frozen MOM.

Cases were subdivided according to the percentages of fresh MOM, PNFMOM and PF fed, as presented in Figure 2. In the fresh MOM group, 54 infants received more than 50% MOM and 40 infants were given pure MOM without PF. In the PNFMOM group, 56 infants received more than 80% PNFMOM, among which 29 received only PNFMOM.

### 3.2 Comparison of clinical outcomes between the fresh MOM and PNFMOM groups

The fresh MOM group had a higher survival rate without severe complications than the PNFMOM group (*p* = 0.010); however, no difference in the mortality rates of the groups was detected. The fresh MOM group also had a significantly lower incidence of LOS and BPD than the PNFMOM group (*p* = 0.033, *p* = 0.022, respectively), but no differences in the incidence of NEC (at least stage 2), ROP were observed between the two groups (Table 2). Table 2 also presents the neonatal outcomes related to nutrition and growth. Compared with the PNFMOM group, the fresh MOM group had a shorter time to regain birth weight (*p* = 0.021), a shorter time to reach total enteral feeding (*p* = 0.024), and a shorter duration of TPN (*p* = 0.045). No differences in feeding intolerance and PNAC were noted between the two groups. No significant differences in growth velocities were observed between the two groups.

**Table 2:** Comparison of outcomes of preterm infants in the fresh MOM and PNFMOM groups

Outcomes	Fresh MOM group (*n* = 82)	PNFMOM group (*n* = 75)	*χ* ^*2*^ value	*p* Value
Neonatal outcomes				
Mortality, *n* (%)	4 (4.9)	4 (5.3)	0.000	1.000
Survival rate without severe complications, *n* (%)	58 (70.7)	38 (50.6)	6.638	0.010
NEC ≥ Stage 2, *n* (%)	8 (9.7)	12 (16.0)	1.374	0.241
LOS, *n* (%)	6 (7.3)	14 (18.7)	4.539	0.033
BPD, *n* (%)	17 (20.7)	28 (37.3)	5.280	0.022
Moderate‐to‐severe BPD, *n* (%)	9 (11.0)	13 (17.3)	1.314	0.252
ROP, *n* (%)	30 (36.6)	33 (44.0)	0.896	0.344
ROP requiring treatment, *n* (%)	4 (4.9)	4 (5.3)	0.000	1.000
Nutritional outcomes				
Time to regain birth weight (days), mean ± SD	6.9 ± 2.0	7.5 ± 1.3	2.328	0.021
Age at total enteral feeding^a^ (days), mean ± SD	25.5 ± 5.9	27.7 ± 6.0	2.284	0.024
TPN (days), mean ± SD	24.3 ± 6.0	26.4 ± 6.9	2.020	0.045
Feeding intolerance, *n* (%)	18 (22.0)	21 (28.0)	0.768	0.381
PNAC, *n* (%)	10 (12.2)	14 (18.7)	1.267	0.260
Growth outcomes				
Weight velocity (g/kg/day), mean ± SD	16.72 ± 1.64	16.97 ± 1.58	0.954	0.342
Length velocity (cm/week), mean ± SD	0.95 ± 0.32	0.91 ± 0.35	0.714	0.476
HC velocity (cm/week), mean ± SD	0.72 ± 0.11	0.68 ± 0.10	1.949	0.053

Abbreviations: BPD, bronchopulmonary dysplasia; HC, head circumference; LOS, late‐onset sepsis; MOM, mother's own milk; NEC, necrotizing enterocolitis; PNAC, parenteral nutrition‐associated cholestasis; PNFMOM, pasteurized never‐frozen MOM; ROP, retinopathy of prematurity; SD, standard deviation; TPN, total parenteral nutrition.

^a^ Excluding cases of death.

Table 3 presents the results concerning survival rate without severe complications, mortality and morbidity with and without adjustments for confounding factors, including gestational age, sex, SGA, any antenatal corticosteroid, surfactant administration, and duration of mechanical ventilation. The survival rate without severe complications was significantly greater in the fresh MOM group with an odds ratio (OR) of 2.606% and a 95% confidence interval (Cl) of 1.21–5.57 (*p* = 0.014). We also observed a reduction in the risk of BPD in the fresh MOM group after adjustment for confounders with an OR of 0.338% and a 95% Cl of 0.14–0.76 (*p* = 0.01). No statistically significant difference was found between the two groups with regard to the risk of LOS after adjustment for confounders.

**Table 3:** Logistic regression analysis of the impact of fresh MOM versus PNFMOM on mortality, survival rate without severe complications and morbidity

Outcomes	Bivariate models		Multivariate models	
	OR	*p* Value	OR	*p* Value
Mortality, *n* (%)	0.91 (0.21–3.77)	0.897	0.896 (0.18–4.30)	0.891
Survival rate without severe complications, *n* (%)	2.35 (1.22–4.53)	0.011	2.590 (1.22–5.49)	0.013
NEC ≥ Stage 2, *n* (%)	0.56 (0.21–1.47)	0.245	0.551 (0.20–1.52)	0.250
LOS, *n* (%)	0.34 (0.12–0.94)	0.039	0.392 (0.13–1.11)	0.079
BPD, *n* (%)	0.38 (0.18–0.78)	0.009	0.345 (0.15–0.77)	0.010
Moderate‐to‐severe BPD, *n* (%)	0.58 (0.23–1.46)	0.255	0.436 (0.14–1.30)	0.139
ROP, *n* (%)	0.53 (0.27–1.01)	0.055	0.541 (0.27–1.05)	0.071
ROP requiring treatment, *n* (%)	0.91 (0.21–3.77)	0.897	1.02 (0.19–5.36)	0.974

*Note*: Reference group = pasteurized MOM. Variables included in the models are as follows: gestational age, sex, SGA, any antenatal corticosteroid, surfactant administration and duration of mechanical ventilation.

Abbreviations: BPD, bronchopulmonary dysplasia; LOS, late‐onset sepsis; MOM, mother's own milk; NEC, necrotizing enterocolitis; OR, odds ratios (Cl 95%); PNFMOM, pasteurized never‐frozen MOM; ROP, retinopathy of prematurity; SGA, small for gestational age.

### 3.3 Feasibility of feeding fresh MOM to very preterm infants

A total of 171 of 199 eligible infants were enrolled in this prospective cohort study. Among the included cases, 56.1% (96/171) of mothers agreed to provide fresh MOM, and the other 75 infants received PNFMOM only. In the fresh MOM group, 14 patients were removed from the study because eight mothers could not provide at least one feeding of fresh milk per day and in six cases the CMV‐DNA levels in fresh MOM exceeded 7 × 10^3^ copies/ml. Thus, 85.4% (82/96) of infants in the fresh MOM group completed the study. Of these, 67.1% (55/82) of premature infants received fresh MOM for more than 50% of enteral feedings.

None of the infants had adverse events related to MOM feeding during the study, including infection caused by breast milk contamination, feeding errors or any other adverse condition caused by clinicians. High levels of CMV‐DNA were detected in 10 MOM samples (4 in the PNFMOM group and 6 in the fresh MOM group), and all 10 samples were then pasteurized. None of the infants in either group were infected with CMV.

## 4 DISCUSSION

In the present study, 56.1% of mothers agreed to provide fresh MOM and 85.4% of infants in the fresh MOM group completed the study. Our results revealed that the fresh MOM without any process group had a higher survival rate without severe complications, lower incidence of LOS and BPD, a shorter time to regain birth weight, a shorter time to reach total enteral feeding, and a shorter duration of TPN than the PNFMOM group, with no statistical difference in mortality, incidence of NEC (at least stage 2), ROP, and growth velocities between the two groups. No adverse events associated with fresh MOM feeding were reported, including CMV infection. These findings suggested that fresh MOM for very preterm infants was feasible and can improve the health of very preterm infants.

Human breast milk is highly regarded as the optimum source of feeding for newborns due to its ability to provide complete nutrition and capacity to confer health factors to the infant. The American Academy of Paediatrics (2012) recommends exclusive breastfeeding for infants. More recently, Witkowska‐Zimny and Kaminska‐El‐Hassan (2017) reported the existence of pluripotent stem cells within fresh human breast milk and demonstrated that human breast milk stem cells can produce self‐renewing stem cells, with a multilineage differentiation potential for all three germ layers, and improve growth and replenish and restore damaged tissues within infants, especially preterm infants. However, a previous study demonstrated that CMV infections can be acquired from fresh breast milk (Vochem et al., 1998). Pasteurization of human milk is a commonly used approach to prevent transmission of potential pathogens such as CMV, but this approach is controversial and guidelines vary. While pasteurization eliminates pathogens, it also reduces concentrations of biologically active components and nutrients (Picaud et al., 2018).

Additionally, although the difference was not statistically significant, which may be due to the small sample size or the fresh MOM feeding protocol, the fresh MOM group tended to have a lower incidence of NEC and LOS than the PNFMOM group. Multiple studies have reported that high‐dose feedings of MOM reduce the incidence, severity and risk of potentially preventable morbidities including NEC (Meier et al., 2017; Quigley et al., 2019) because the process of pasteurization reduces the antioxidant properties of human milk (Juncker et al., 2021). A previous randomized controlled trial (RCT) in Canada reported that the NEC ≥ Stage 2 rate was only 1.7% in mother milk‐fed infants supplemented with donor milk compared with 6.6% in infants who received supplemental formula, which was much lower than our data (O'connor et al., 2016). However, our results are consistent with the previous study in China. An intervention cohort study in China (Q. Zhou et al., 2020) showed that the NEC (≥Stage 2) rates were 7.55% in the MOM group, and a multicentre prospective cohort study (Sun et al., 2019) showed that the NEC (≥Stage 2) rates were 6% in the MOM group and 14% in the frozen MOM group. Possible reasons why the NEC rates were relatively high are as follows: first, there were more mothers who did not provide MOM at the early feeding stage, and thus, formula was given early enough. Further, the time point of weaning was different for infants, and therefore, the feeding time was varied. Because the LOS rate was high in the PNFMOM group, prolonged total enteral feeding and TPN use can cause intestinal flora disorder.

Our study demonstrated a significant reduction in the BPD risk with the feeding of fresh MOM. Consistently, Patel et al. (2017) reported a 9.5% reduction in the odds of BPD for every 10% increase in the dose of MOM. It has been suggested that BPD, ROP, NEC and patent ductus arteriosus are all forms of oxygen radical disease (Hård et al., 2019; Spiegler et al., 2016). Previous studies reported that fresh human milk has the highest antioxidant activity, and freezing and pasteurization reduce the total antioxidant capacity of breast milk to varying degrees (Păduraru et al., 2018).

In the present study, infants fed fresh MOM regained birth weight earlier, achieved total enteral feeding earlier and required a shorter duration of TPN compared with those fed PNFMOM. Păduraru et al. showed that gastric emptying was slightly faster in preterm infants fed with fresh human milk than in those fed with pasteurized human milk (Perrella et al., 2015). This suggests that fresh human milk is better digested and/or promotes greater intestinal motility in premature infants as it retains the greatest nutritional components, while pasteurization inactivates bile salt‐stimulated lipase, thus decreasing fat absorption and reducing milk levels of insulin and insulin‐like growth factors, which are thought to stimulate gut maturation and growth (Koh et al., 2020). However, no difference in growth was observed between the two groups in the present study, consistent with the results of previous observational studies (Cossey et al., 2013; Dicky et al., 2017). This can likely be attributed to the fact that both groups received different amounts of PF, and more weight is gained with formula feeding, as described by Spiegler et al. (2016). It is possible that fresh MOM is not enough to affect the early growth of the infants, and the ideal model for exclusive fresh MOM feeding to promote growth needs to be determined in future research.

Because the only six samples with CMV‐DNA levels above 7 × 10^3^ copies/ml in the fresh MOM group were pasteurized, we did not assess the safety of fresh MOM in terms of CMV. This needs to be investigated in future studies.

## 5 STRENGTH AND LIMITATIONS

The strengths of the present study include its prospective cohort design and 2‐year study period, with a follow‐up at least 36 weeks after birth. We included only cases fed with fresh MOM without cooling, freezing, pasteurizing or heating, which was almost equal to breastfeeding. Furthermore, many related outcomes and indexes were compared, such as the survival rate without severe complications, LOS, NEC and others not only in relation to the main outcome but also nutritional and growth outcomes.

There are some limitations to the current study. First, this was a single‐centre study, which limits the generalization of the results, and the sample size was not large enough to adequately assess the association of fresh MOM with morbidities that occurred at low rates, such as NEC or ROP. Second, we are unable to assess the more detailed dose effects of fresh MOM because some infants were fed a combination of fresh MOM, pasteurized MOM and PF. Future studies with larger cohorts and documented amounts of MOM feedings or RCTs are needed to calculate possible dose effects.

## 6 CONCLUSIONS

In summary, compared to very preterm infants fed PNFMOM, those infants fed fresh MOM advanced more rapidly to full enteral nutrition, required a shorter duration of parenteral nutrition and demonstrated a higher survival rate without severe complications as well as a lower incidence of BPD even with adjustment for confounders. This study indicates that fresh MOM feeding is a feasible feeding strategy to improve outcomes among preterm infants. The results of this study should be considered in the debate about whether to pasteurize MOM and can inform feeding guidelines for these particularly vulnerable infants. More large, multicenter studies are needed to confirm the findings of this study and establish optimal feeding guidelines for very preterm infants.

## AUTHOR CONTRIBUTIONS

**Jing Huang:** Conceptualization; data curation; formal analysis; methodology; project administration; resources; software; roles/writing – original draft. **Xiao‐lan Zhang and Xin‐zhu Lin:** Conceptualization; funding acquisition; validation; visualization; writing – review and editing. **Zhi Zheng and Xiao‐yan Zhao:** Investigation; project administration; resources; software; supervision. **Li‐han Huang and Lian Wang:** Data curation; formal analysis.

## CONFLICT OF INTEREST

The authors declare no conflict of interest.

## ETHICS STATEMENT

The ethics committee of Xiamen Maternal and Child Care Hospital for human research approved the study (approval no. KY‐2016‐020). All procedures involving human participants were performed in accordance with the ethical standards of the institutional and national research committee and with the 1964 Declaration of Helsinki and its later amendments or comparable ethical standards. The parents of all study participants provided written informed consent.

## Data Availability

The data sets generated and analysed during the current study are available from the corresponding author upon reasonable request.
